# Unusual Localization of Blood-Borne *Loa loa* Microfilariae in the Skin Depends on Microfilarial Density in the Blood: Implications for Onchocerciasis Diagnosis in Coendemic Areas

**DOI:** 10.1093/cid/ciab255

**Published:** 2021-06-14

**Authors:** Yannick Niamsi-Emalio, Hugues C Nana-Djeunga, Cédric B Chesnais, Sébastien D S Pion, Jules B Tchatchueng-Mbougua, Michel Boussinesq, María-Gloria Basáñez, Joseph Kamgno

**Affiliations:** 1 Centre for Research on Filariasis and other Tropical Diseases (CRFilMT), Yaoundé, Cameroon; 2 Institut de Recherche pour le Développement (IRD), UMI233/INSERM U1175, Université de Montpellier , Montpellier, France; 3 Service d’Epidémiologie, Centre Pasteur du Cameroun, Membre du Réseau International des Instituts Pasteur, Yaoundé, Cameroun; 4 MRC Centre for Global Infectious Disease Analysis and London Centre for Neglected Tropical Disease Research, Dep artment of Infectious Disease Epidemiology, School of Public Health, Imperial College London, London, United Kingdom; 5 Faculty of Medicine and Biomedical Sciences, University of Yaoundé I, Yaoundé, Cameroon

**Keywords:** *Onchocerca volvulus*, *Loa loa*, diagnosis, classification and regression tree model, skin snip technique

## Abstract

**Background:**

The diagnostic gold standard for onchocerciasis relies on identification and enumeration of (skin-dwelling) *Onchocerca volvulus* microfilariae (mf) using the skin snip technique (SST). In a recent study, blood-borne *Loa loa* mf were found by SST in individuals heavily infected with *L. loa*, and microscopically misidentified as *O. volvulus* due to their superficially similar morphology. This study investigates the relationship between *L. loa* microfilarial density (*Loa* MFD) and the probability of testing SST positive.

**Methods:**

A total of 1053 participants from the (onchocerciasis and loiasis coendemic) East Region in Cameroon were tested for (1) *Loa* MFD in blood samples, (2) *O. volvulus* presence by SST, and (3) Immunoglobulin (Ig) G4 antibody positivity to Ov16 by rapid diagnostic test (RDT). A Classification and Regression Tree (CART) model was used to perform a supervised classification of SST status and identify a *Loa* MFD threshold above which it is highly likely to find *L. loa* mf in skin snips.

**Results:**

Of 1011 Ov16-negative individuals, 28 (2.8%) tested SST positive and 150 (14.8%) were *L. loa* positive. The range of *Loa* MFD was 0–85 200 mf/mL. The CART model subdivided the sample into 2 *Loa* MFD classes with a discrimination threshold of 4080 (95% CI, 2180–12 240) mf/mL. The probability of being SST positive exceeded 27% when *Loa* MFD was >4080 mf/mL.

**Conclusions:**

The probability of finding *L. loa* mf by SST increases significantly with *Loa* MFD. Skin-snip polymerase chain reaction would be useful when monitoring onchocerciasis prevalence by SST in onchocerciasis–loiasis coendemic areas.

Onchocerciasis is a parasitic infection caused by the filarial nematode *Onchocerca volvulus* and transmitted by *Simulium* blackfly vectors. Sessile adult female worms live inside nodules or worm bundles, where they mate with the (mobile) males and periodically produce thousands of embryos or microfilariae [1]. Microfilariae (mf) leave the nodules to populate the skin under the epidermis and the eyes and may live for a maximum of 2.5 years [1]. Most (99%) onchocerciasis cases occur in sub-Saharan Africa. The infection is associated with skin pathology, ocular lesions that progressively lead to blindness, excess mortality, and epilepsy [2–5]. The severity of these manifestations depends on the burden of infection, particularly on (past and/or present) *O. volvulus* microfilarial density (MFD) [6–9].

In endemic countries, regional control and elimination initiatives have been implemented, based on vector control and/or mass drug administration (MDA) with ivermectin. Elimination of transmission (EOT) has been achieved in formerly endemic Latin American countries (Mexico, Guatemala, Colombia, Ecuador) under the auspices of the Onchocerciasis Elimination Program for the Americas (1993–present) [10], with the exception of the Amazonian focus straddling Venezuela and Brazil [11]. In Africa, the Onchocerciasis Control Programme in West Africa (OCP; 1974–2002) and the African Programme for Onchocerciasis Control (APOC; 1995–2015) led to substantial reductions in disease burden [12] and to EOT in some foci of West and East Africa [13, 14]. Levels of precontrol endemicity and progress towards morbidity and EOT targets have been measured via assessment of prevalence and intensity of infection: in the OCP by detection and enumeration of skin mf by the skin snip technique (SST) [15] and in APOC by nodule prevalence at the beginning of the program and by SST for phase 1A (monitoring prevalence decline) and phase 1B (stop-MDA) evaluations [16]. The SST consists of taking 2 or more bloodless skin snips (typically with a Holth-type corneoscleral punch), incubating the snips in a suitable medium (eg, saline) for 24 hours, and detecting the presence (for prevalence) and number (for MFD) of emerged mf in the medium under a microscope [15].

However, the microfilarial morphology of *Loa loa* (another *Chrysops-*transmitted, filarial infection endemic in West and Central Africa) is superficially similar to that of *O. volvulus* (albeit *L. loa* mf are sheathed while those of *O. volvulus* are not) when preparations are not stained, making it difficult to distinguish between these 2 species using bright-field microscopy (*L. loa* mf measure 250‒300 μm by 6‒8 μm and those of *O. volvulus* measure 220‒360 µm by 5‒9 µm). Since *L. loa* mf have been considered to be solely blood-dwelling, the presence of *L. loa* mf in skin snips has seldom been investigated. A recent study demonstrated the occurrence of *L. loa* mf when using SST and discussed its implications for potential reporting of *O. volvulus* false positives [17]. Not only would this impact individual diagnosis but also epidemiological evaluations of interventions. Therefore, this study aimed to analyze the relationship between *L. loa* MFD in the blood (mf/mL) and the probability of detecting *L. loa* mf in skin snips during onchocerciasis diagnosis in an area where infections by *O. volvulus* and *L. loa* are coendemic.

## METHODS

### Ethical Approval

The study received ethical clearance from the Cameroon National Ethics Committee for Research for Human Health (CNERSH; no. 2015/01/545/CE/CNERSH/SP). The survey was approved by and undertaken under the authority of the Ministry of Public Health of Cameroon following the Helsinki Declaration. Participation in this study was entirely voluntary and refusal to participate had no consequence for individuals. The protocol (objectives, methodology, use of collected data, and dissemination of results) was carefully explained to all eligible individuals. Those who agreed to participate signed an informed-consent form before undergoing clinical examination and sample collection. Parents or legal guardians provided their approval upon enrollment of minors (aged <21 years).

### Study Area and Design

Data were collected during a study conducted in the East Region of Cameroon in March 2015 for integrated remapping of onchocerciasis, lymphatic filariasis, loiasis, malaria, and soil-transmitted helminthiases, which are coendemic in this region [18, 19]. Since 2004, community-directed treatment with ivermectin (CDTI) for onchocerciasis has been implemented in 3 health districts (HDs)—namely, Bertoua, Betare Oya, and Lomie—out of the 14 HDs in the East Region.

For epidemiological monitoring, the East Region has been organized into 5 zones or evaluation units (EUs). A cross-sectional survey was conducted in a community selected in each of these EUs. Individuals of both sexes, aged 5 or more years, and residing in the selected community were eligible for the study and invited to participate. At least 150 individuals in each selected community were sampled.

### Parasitological and Serological Assessment

*Loa loa* MFD (*Loa* MFD) was quantified using calibrated thick blood smears. A sample of 50 µL of peripheral blood was collected by finger-prick between 10 am and 2 pm (due to diurnal periodicity of *L. loa* mf [20]). After de-hemoglobinization and Giemsa staining, the slides were examined using bright-field microscopy. All mf present on the slide, including *L. loa* and *Mansonella perstans*, were identified, counted, and expressed as mf/mL. Here we only report *Loa* MFD.

Onchocerciasis was assessed by 2 different methods—namely, the SST to detect mf in the skin and a rapid diagnostic test (RDT)—to detect the presence of immunoglobulin G4 (IgG4) antibodies against the *O. volvulus*–specific antigen Ov16 [21]. For SST, 2 skin biopsies (one from each posterior iliac crest) were taken using a 2-mm Holth-type corneoscleral punch and incubated for 24 hours in saline; the presence of emerged mf was assessed under an optical microscope. Antibody testing was done using the Standard Diagnostics (SD) Bioline biplex Ov16/Wb123 RDT as per the manufacturer’s instructions, with specificity listed at 100%. Recently published studies using the biplex RDT [22] and studies using earlier prototypes have reported a specificity of 97.5% [23].

### Dependent Variable

The dependent variable was a false-positive (+/ve) result for SST, defined as the presence of mf under the microscope whilst testing negative (–/ve) for Ov16 RDT. Combining the results of the 2 tests produced 4 categories: (1) active *O. volvulus* infections (SST+/ve and RDT+/ve), (2) true negatives (SST–/ve and RDT–/ve), (3) likely past infections (SST–/ve but RDT+/ve), and (4) false positives (SST+/ve but RDT–/ve).

### Statistical Modeling Analysis

The results for the categorical variables (sex, community, SST status, Ov16 RDT status) are presented as proportions. The results for the numerical variables (age, *Loa* MFD) are summarized by medians and their interquartile range (IQR).

A logistic regression model was used to assess associations between SST false positivity and the following covariates: individuals’ age and sex, community of residence, and *Loa* MFD. The covariate *Loa* MFD was introduced in the model as a fractional polynomial [24] to capture possible nonlinear associations. The choice of polynomial powers was based on minimizing the deviance information criterion (DIC) of the model. The Akaike information criterion (AIC) [25, 26] was also used to select the best-fit model (that with the lowest AIC value). The predicted probability of SST false positivity was estimated using the function “predict” of the R software [27] using the best-fit regression model.

A Classification and Regression Tree (CART) model (based on an iterative algorithm) was used to estimate the covariates associated with SST false positivity. CART is a nonparametric multiple regression statistical model to perform supervised classifications with regard to explanatory variables compared with a categorical variable [28, 29]. The modeling is carried out in 3 stages: (1) identification of the covariates associated with the response variable, (2) classification of each covariate to discriminate the response variable into 2 distinct groups, and (3) iterative repetition of the 2 previous steps until it is no longer possible to perform segmentation [30]. The 95% confidence interval (CI) for the identified threshold was obtained by bootstrapping [31], based on the percentile method with 10 000 replicates. In order to assess the difference in risk of being SST false positive between different *Loa* MFD classes, a logistic regression model was implemented to quantify, through the calculation of odds ratios (ORs) and their 95% CIs, the association between the probability of false positivity and covariates (age, sex, community, *Loa* MFD).

All analyses were performed with R version 3.6.1 using the packages MFP (multiple fractional polynomial) and ggplot2 [27, 31]. The *P* value used for statistical significance was *P* < .05.

## RESULTS

A total of 1053 (19.8%) individuals aged 6–85 (median, 28; IQR, 12–48) years participated in the study (out of 5314 individuals residing in the 5 study communities). The number of participants per community ranged between 156 and 318, with a sex ratio generally female biased (Table 1).

**Table 1.** Description of the Population Tested for *Loa loa* and *Onchocerca Volvulus* in 5 Communities of the East Region, Cameroon

Community	No. of Participants (% of Total)	Sex Ratio (F/M)	Median (IQR) Age, Years	No. (%) With *L. loa* mf	No. (%) SST Positive	No. (%) Ov16 RDT Positive	No. (%) Ov16 RDT Negative but SST Positive
Adjela	156 (13.8)	1.7 (99/57)	26.0 (13.0–51.3)	8 (5.1)	11 (7.1)	16 (10.3)	5 (3.2)
Azemkout	318 (31.1)	1.4 (186/132)	15.0 (10.0–44.0)	45 (14.2)	11 (3.5)	4 (1.3)	10 (3.1)
Mikel	232 (22.5)	2.0 (155/77)	25.0 (11.8–42.0)	31 (13.4)	3 (1.3)	5 (2.2)	3 (1.3)
Pana	175 (16.7)	1.4 (102/73)	30.0 (15.0–50.5)	52 (29.7)	6 (3.4)	6 (3.4)	6 (3.4)
Timangolo	172 (15.9)	0.6 (65/107)	40.0 (24.0–53.3)	22 (12.8)	6 (3.5)	11 (6.4)	4 (2.3)
All	1053 (19.8)	1.4 (607/446)	28.0 (12.0–48.0)	158 (15.0)	37 (3.5)	42 (4.0)	28 (2.7)

Abbreviations: F, female; IQR, interquartile range; M, male; mf, microfilariae; Ov16 RDT, Ov16 rapid diagnostic test; SST, skin snip technique.

The between-community prevalence of *L. loa* microfilaremia varied from 5.1% (95% CI, 2.2–9.8%) to 29.7% (95% CI, 23.1–37.1%). The SST revealed that 37 (3.5%) individuals presented with skin mf, while the Ov16 RDT revealed 42 (3.4%) positive cases. Among the 1011 Ov16-negative individuals, 28 (2.8%) tested SST positive and were considered as false positives. Of these, 21 (75%) presented with blood *L. loa* mf. The range of *Loa* MFD among the 28 false positives was 0–85 200 mf/mL.

The characteristics of the 28 SST false-positive individuals are shown in Supplementary Table 1. Although not statistically significant in the univariate analysis (*P* = .070), the number of false positives was approximately 3 times higher among females (21) than males (7). Likewise, the number of SST false positives was nearly double in individuals aged older than 40 years (18) compared with those aged 40 years or younger (10) (*P* < .001). The median *Loa* MFD was 5940 (IQR, 450–13 820) mf/mL among the SST positives and 0 (IQR, 0–0) among the SST negatives (*P* < .001). The proportions of SST false positives were similar between communities (*P* = .603) (Table 2). Multivariate analysis (Supplementary Table 2) revealed that SST false positivity was significantly and negatively associated with male sex (*P* = .0226) and positively with *Loa* MFD (*P* < .0001).

**Table 2.** Univariate Association Between Skin Snip Technique False Positivity (for *Onchocerca Volvulus*) and Sex, Age, *Loa loa* Microfilarial Density, and Community of Residence Among Ov16-Negative Individuals

Variable	Values	*P*
Intercept		…
Sex, n/N (%)		.070
Female	21/590 (3.6)	
Male	7/421 (1.7)	
*Loa* MFD, mf/mL		
Median	5940	<.001
IQR	(450–13 820)	
Age, n/N (%)		<.001
5–40 years	10/682 (1.5)	
41–85 years	18/329 (5.5)	
Community, n/N (%)		.6033
Adjela	5/140 (3.6)	
Azemkout	10/314 (3.2)	
Mikel	3/227 (1.3)	
Pana	6/169 (3.6)	
Timangolo	4/161 (2.5)	

N = 1011. Abbreviations: IQR, interquartile range; *Loa* MFD, *Loa loa* microfilarial density (mf/mL); mf, microfilariae.

### Predictors and Predicted Probability of Being False Positive for Onchocerciasis

Several models were run to select the best one according to the AIC and DIC criteria. The logistic model with fractional polynomial (for *Loa* MFD) was the best-fit model (lowest AIC and DIC) (Supplementary Table 3). Since age and *Loa* MFD were positively and statistically significantly associated (*P* < .0001), age was not included in the multivariate analysis. Males were less likely than females to be SST false positive (*P* = .023). The probability of being SST false positive increased with *Loa* MFD (*P* < .001).

**Figure 1.**
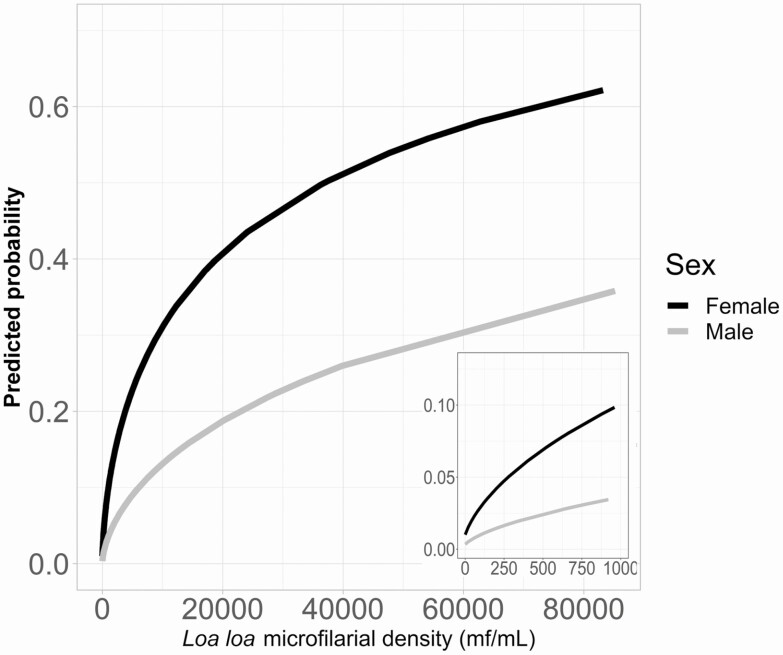
Predicted probability of SST false positivity for females (black solid line) and males (gray solid line) as a function of *Loa* MFD. The inset depicts the relationship for *Loa* MFD <1000 mf/mL. Abbreviations: *Loa* MFD, *Loa loa* microfilarial density; mf, microfilariae; SST, skin snip technique.

Figure 1 illustrates the predicted probability of being SST false positive as a (nonlinear) function of *Loa* MFD according to sex. For both sexes, the probability of being SST false positive increases steeply for low *Loa* MFD values, with the rate of increase slowing down for high *Loa* MFD values. For a given *Loa* MFD, females were consistently more likely to be SST false positive than males. Supplementary Table 4 presents values of predicted probabilities of being SST false positive according to *Loa* MFD.

### Classification of Ov16 Negatives According to *Loa* MFD

**Figure 2.**
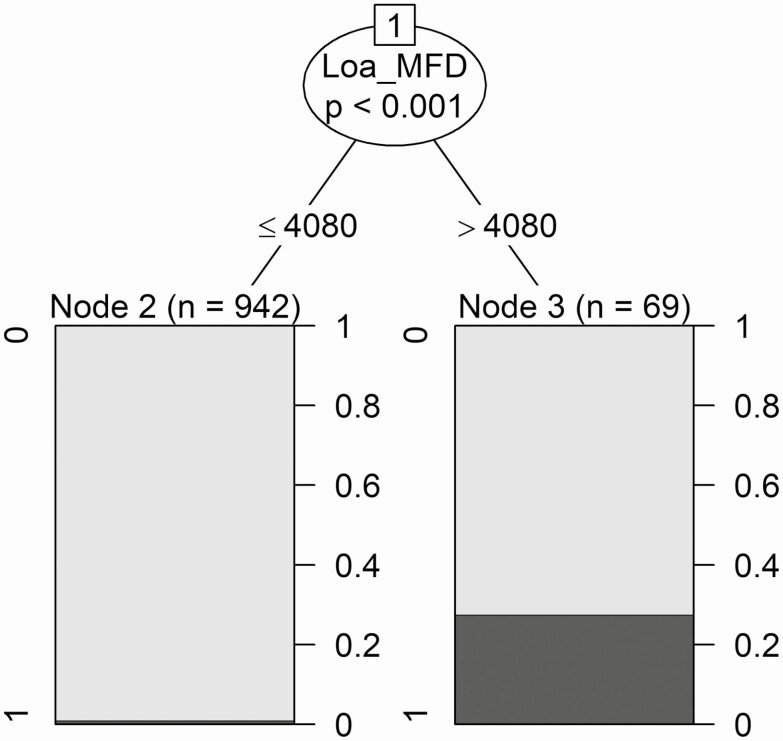
Results of the CART model for the 1011 Ov16-negative individuals according to SST status. Dark-gray bars indicate positive SST and gray bars indicate negative SST. Class A (node 2 on the left branch) corresponds to (942) individuals with *Loa* MFD ≤ 4080 mf/mL; class B (node 3 on the right branch) corresponds to (69) individuals with *Loa* MFD >4080 mf/mL. The right-hand vertical axes indicate the probability of being SST false positive in the 2 classes. Abbreviations: CART, Classification and Regression Tree; *Loa* MFD, *Loa loa* microfilarial density; mf, microfilariae; SST, skin snip technique.

The CART model identified that a single value of *Loa* MFD, estimated at 4080 (95% CI, 2180–12 240) mf/mL, allowed discrimination of the 1011 Ov16-negative individuals into 2 classes. Class A included 942 individuals with *Loa* MFD of 4080 mf/mL or less; the probability of being SST false positive was 1%. Class B had 69 individuals with *Loa* MFD greater than 4080 mf/mL; the probability of being SST false positive was 27.5% (Figure 2). The distribution of the sexes among these 2 classes was similar (*P* = .183).

**Figure 3.**
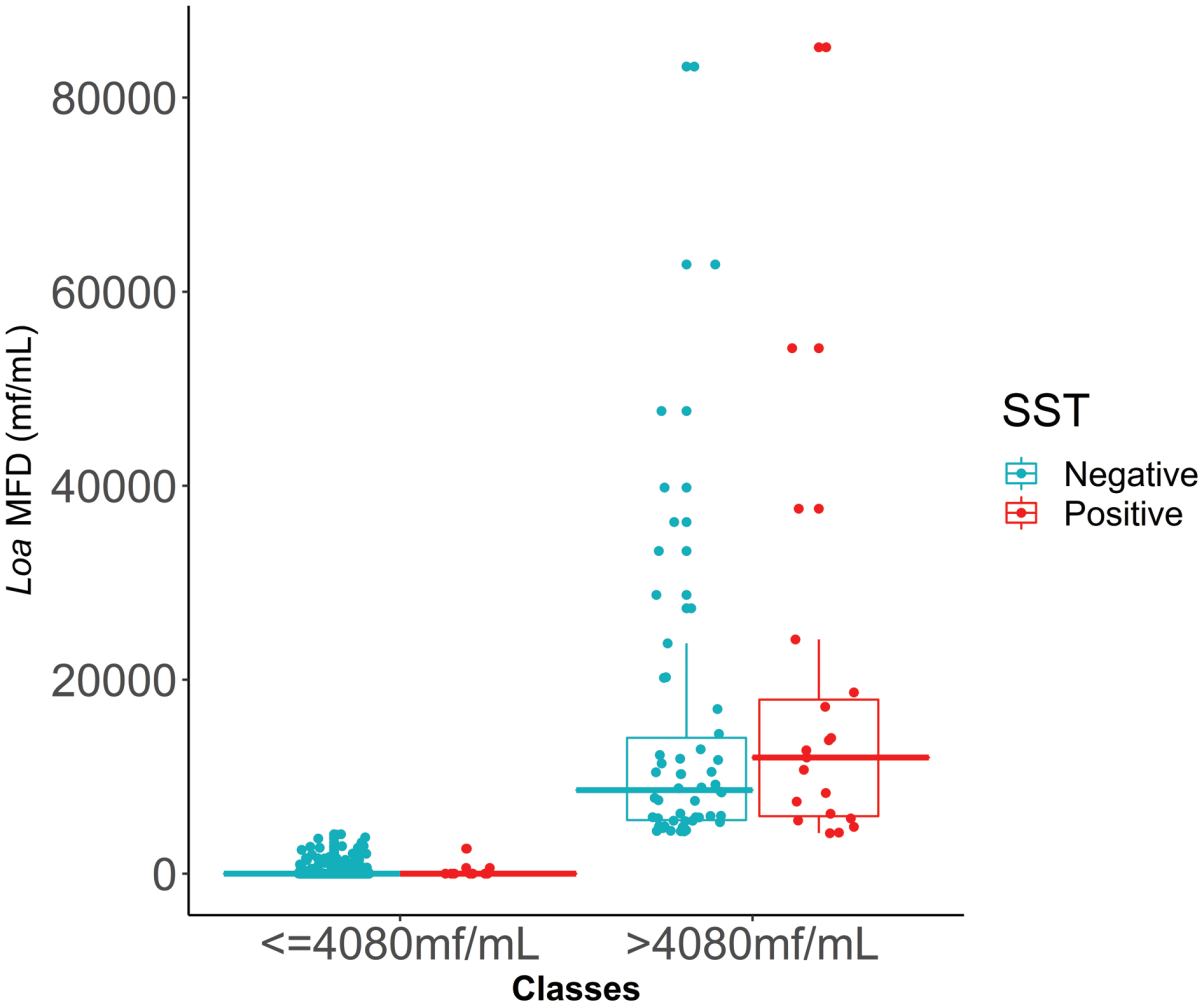
Distribution of individual *Loa* MFD values according to the threshold of 4080 mf/mL and SST results in the 1011 Ov16-negative individuals. The 2 classes (≤4080 mf/mL and >4080 mf/mL) are those obtained with the CART model illustrated in Figure 2. Turquoise solid circles denote SST negatives; red solid circles represent SST positives (9 individuals with *Loa* MFD below the threshold and 19 individuals with *Loa* MFD above the threshold). The solid horizontal lines within the boxes are the medians; the lower and upper borders of the boxes are, respectively, the first and third quartiles (IQR, Q3–Q1); the vertical bars indicate the “minimum” and “maximum” calculated as Q1 – 1.5 × IQR and Q3 + 1.5 × IQR, respectively. Abbreviations: CART, Classification and Regression Tree; IQR, interquartile range; *Loa* MFD, *Loa loa* microfilarial density; mf, microfilariae; Q, quartile; SST, skin snip technique.

Figure 3 shows the distribution of individual *Loa* MFD values for the 1011 Ov16-negative individuals according to the threshold of 4080 mf/mL and whether they tested SST negative or positive. Most SST positives were found in class B and exhibited high *Loa* MFD. Class B individuals were significantly more likely to be false SST positive than class A individuals (OR, 46.7; *P* < .001). Males were less likely to be false SST positive compared with females, regardless of *Loa* MFD category (OR, 0.3; *P* = .013) (Table 3).

**Table 3.** Association Between *Loa loa* Microfilarial Density and Testing Positive With the Skin Snip Technique for Ov16-Negative Individuals

Variables	OR (95% CI)	95% CI (Bootstrap)	*P*
Intercept	.013 (.00–.03)	(.00–.02)	…
*Loa* MFD class			
A: ≤4080 mf/mL	1		…
B: >4080 mf/mL	46.7 (20.1–116.8)	(20.6–138.0)	<.001
Sex			
Female	1		…
Male	.3 (.1–.7)	(.1–.7)	.013

N = 1011. Abbreviations: CI, confidence interval; *Loa* MFD, *Loa loa* microfilarial density (mf/mL); mf, microfilariae; OR, odds ratio.

## Discussion

One of the challenges facing onchocerciasis control and elimination program managers and their partners is the availability of reliable diagnostic tools that can be used to assess infection trends during the treatment phase to be able to make stopping ivermectin MDA decisions and transition to post-treatment surveillance. The tests generally used in these contexts are the SST (to monitor progress during the treatment/intervention phase [16]), the Ov16 serology test (to measure exposure, particularly in children younger than 10 years), and the *O. volvulus* DNA (O-150 polymerase chain reaction [PCR] pool screen) test in blackflies (the latter 2 to confirm transmission interruption and stop MDA [32–34]). A major disadvantage of Ov16 serology is that it cannot differentiate between active and past *O. volvulus* infection. Also, it has been suggested that approximately 20% of individuals in endemic communities may be unable to mount an IgG4 antibody response to Ov16 [35], resulting in a false-negative outcome. In addition, there is a multiplicity of Ov16 assays, including various versions of the enzyme-linked immunosorbent assay and the RDT used here, which have different sensitivity and specificity characteristics, and this may vary between laboratories or between these and field settings [34]. In the case of the biplex RDT used here, we accepted the value of 100% specificity given by the manufacturers [34, 36] but are aware that others have reported a lower (97.5%) specificity [22, 23]. We assumed that when the RDT test was negative, an individual with an SST+/ve result would be a false positive for *O. volvulus*. Given that our overall proportion of false positives was 2.7%, our results should be interpreted with caution.

The SST has been considered as the “gold standard” benchmark to detect and quantify *O. volvulus* microfilaridermia. However, its sensitivity decreases during control programs because *O. volvulus* MFD declines as a result of CDTI, and although SST sensitivity can be improved by increasing the number of snips taken [37], this may not be practical or acceptable. Besides, in areas of coendemicity with skin-dwelling *Mansonella streptocerca* (endemic to Africa, mf measuring 180‒240 µm by 3‒5 µm), or blood-dwelling *M. perstans* (200 µm by 4.5 µm), *Mansonella ozzardi* (coendemic with *O. volvulus* in the extant Amazonian focus, mf measuring 160‒205 µm by 4 µm) [38], and *L. loa* [17], mf of these species can occur in skin snips and be misidentified as *O. volvulus* mf when assessing microfilaridermia by SST. Consequently, there is a risk that in areas where filarial diseases are coendemic, false *O. volvulus* positives can be detected, with repercussions for individual diagnosis and epidemiological evaluations. The aim of this work was, therefore, to determine the relationship between *Loa* MFD and the probability of a positive diagnosis by SST while being negative by Ov16.

Results indicate that the relationship between false positivity to SST and *Loa* MFD is nonlinear and statistically significant. The probability of being false positive to *O. volvulus* by SST increases sharply at low *Loa* MFD and less steeply at high *Loa* MFD values, being consistently higher for females compared with males—that is, for the same MFD, the probability is 2–3 times as high for females as for males (Supplementary Table 4). In the Nana-Djeunga et al [17] study, *O. volvulus* SST positivity was significantly higher in males (who might therefore be less likely to exhibit false positivity). Sex-specific exposure patterns to *O. volvulus* and *L. loa* are likely to be different [33, 39].

The discrimination of the sample through a supervised classification model yielded statistical significance for *Loa* MFD only. Neither age or sex nor community of residence were significant predictors. The CART model produced 2 *Loa* MFD classes, the optimal discrimination value between the 2 being 4080 mf/mL, representing the threshold above which it is very (nearly 30%) likely to find *L. loa* mf in skin snips. This relatively low value reflected the distribution of *Loa* MFD in the individuals examined. Half of the sample had *Loa* MFD less than 6000 mf/mL, the median was 5940 mf/mL, and the 95% CI for the threshold was 2180–12 240 mf/mL. Above 12 240 mf/mL, the probability of being SST false positive was greater than 35% for females and greater than 15% for males, reaching more than 60% for females and approximately 35% for males for *Loa* MFD = 80 000 mf/mL. This result is consistent with the previous study [17], in which the chances of being SST positive increased with *Loa* MFD.

The mechanisms explaining the presence of *L. loa* mf in the skin are yet to be investigated, but potential explanations may include that *L. loa* mf are in the dermal microcapillaries when the skin biopsy is taken. Although skin snips are supposed to be bloodless, this may not always be achieved when using corneoscleral punches, sampling a large number of individuals, and/or having a number of technicians undertaking the procedure who may vary in their skill to perform the SST. Importantly, the association of this phenomenon with increasing microfilaremia levels is of interest, and similar to what has been described with *O. volvulus* mf, which may be found in blood when skin MFDs are high [40]. This phenomenon led to the description of *Microfilaria bolivarensis* in heavily infected Amerindian populations of the Amazonian focus [41], which was later demonstrated to be *O. volvulus* in blood [40].

Notwithstanding these considerations, it is increasingly recognized that the skin may be an organ of crucial importance in the transmission of (blood-dwelling) vector-borne parasites (eg, *Trypanosoma brucei gambiense* [42], *Leishmania donovani* [43], and *Plasmodium falciparum* [44]) and this phenomenon should be rigorously investigated in loiasis. We also recommend that further studies be carried out in different epidemiological settings and populations to assess the applicability and variability of the *Loa* MFD threshold identified in this study.

### Conclusions

The diagnosis and epidemiological evaluations of onchocerciasis, particularly at baseline and during early phases of control interventions, rely on the detection and enumeration of *O. volvulus* mf by SST. In areas coendemic with loiasis, *L. loa* mf may also be found in the skin, emerging from dermal arterioles and venules when snips are taken with corneoscleral punches during SST, potentially confounding its results. In onchocerciasis–loiasis coendemic areas, a test-and-not-treat strategy has been developed to prevent post-ivermectin severe adverse events, enabling safe treatment of onchocerciasis. This strategy relies on LoaScope testing to measure *Loa* MFD and to exclude individuals with *Loa* MFD greater than 20 000 mf/mL from ivermectin treatment [45]. In these areas, testing such individuals for onchocerciasis and treating them with anti-*Wolbachia* therapies (eg, doxycycline) provides a safe and appropriate alternative treatment strategy to help eliminate onchocerciasis [46], as *L. loa* lacks *Wolbachia* endosymbionts [47]. This test-and-treat with doxycycline strategy [48] would benefit from using methods such as real-time PCR and loop-mediated isothermal amplification (LAMP) assays for the amplification of *O. volvulus* DNA from skin snips [34]. A next step to investigate the impact of our results on onchocerciasis programs would be the inclusion, in a loiasis transmission dynamics model such as EPILOA [49], of the distribution of *L. loa* microfilarial loads to ascertain the proportion of the population who would likely test false-positive for *O. volvulus* in coendemic areas. The *Loa* MFD threshold (4080 mf/mL) above which the likelihood increases of having an SST false-positive result for onchocerciasis should be further investigated and validated. Should this phenomenon be confirmed, its consequences for disease manifestations and onward transmission would warrant further studies.

## Supplementary Data

Supplementary materials are available at *Clinical Infectious Diseases* online. Consisting of data provided by the authors to benefit the reader, the posted materials are not copyedited and are the sole responsibility of the authors, so questions or comments should be addressed to the corresponding author.

ciab255_suppl_Supplementary-Material

## Notes

***Authors’ contributions.*** H. C. N.-D., M. B., M.-G. B., and J. K. conceived and designed the study; Y. N.-E., C. B. C., S. D. S. P., J. B. T.-M., and M.-G. B. analyzed the data and conducted the modeling; Y. N.-E., H. C. N.-D., C. B. C., S. D. S. P., and M.-G. B. wrote the paper. All authors commented on and revised interim and final drafts of the manuscript.

***Acknowledgments.*** We thank the health authorities of the East Region of Cameroon for their support during the study, and particularly the volunteers from the study communities who agreed to take part in the data- and sample-collection phases of this study.

***Supplement sponsorship.*** This article appears as part of the supplement “Sustainable control of neglected tropical diseases – surveillance and diagnostics”, sponsored by the NTD Modelling Consortium.

***Disclaimer.*** The authors alone are responsible for the views, opinions, assumptions, or any other information expressed in this manuscript, which are not necessarily the views, decisions, or policies of the institutions with which they are affiliated.

***Financial support.*** The data collection of this study was co-funded by the Bill & Melinda Gates Foundation (grant number OPP1033740; to M. B.) through the Task Force for Global Health project “Filling the Gaps: Operational Research to Ensure the Success of the Neglected Tropical Disease Control and Elimination Programs” (grant number NTD-SC/NCT 049; to J. K.); the African Programme for Onchocerciasis Control (APOC); and the US Agency for International Development (USAID) ENVISION Project. ENVISION (2011‒2019) was a global project led by RTI International and funded by the USAID (cooperative agreement number AID-OAA-A-11-00048; to J. K.). M.-G. B. and J. K. acknowledge funding from The Royal Society International Exchange Programme (grant number IES\R2\181017). M.-G. B. acknowledges funding of the NTD Modelling Consortium by the Bill & Melinda Gates Foundation (grant number OPP1184344) and joint center funding (grant number MR/R015600/1) by the UK Medical Research Council (MRC) and the UK Department for International Development (DFID) under the MRC/DFID Concordat agreement, which is also part of the European and Developing Countries Clinical Trials Partnership (EDCTP2) program supported by the European Union. J. K. was also funded by Institut Bertrand Buisson, France (subaward number 2011_TNT_01 to the Centre for Research on Filariasis and other Tropical Diseases [CRFilMT]).

***Potential conflicts of interest.*** The authors: No potential conflicts of interest. All authors have submitted the ICMJE Form for Disclosure of Potential Conflicts of Interest. Conflicts that the editors consider relevant to the content of the manuscript have been disclosed.
