# Supplementary material for: Unusual Localization of Blood-Borne Loa loa Microfilariae in the Skin Depends on Microfilarial Density in the Blood: Implications for Onchocerciasis Diagnosis in Coendemic Areas
Source: Clin Infect Dis. 2021 Jun 14;72(Suppl 3):S158–64. doi: 10.1093/cid/ciab255 (PMC8201578; doi:10.1093/cid/ciab255)
Supplement: ciab255_suppl_Supplementary-Material [file ciab255_suppl_supplementary-material.pdf]

Clin Infect Dis Special Collection on Sustainable NTD control – Surveillance and Diagnostics

Supplementary File

Unusual localization of blood-borne *Loa loa* microfilariae in the skin depends on microfilarial density in the blood: Implications for onchocerciasis diagnosis in co-endemic areas

Yannick Niamsi-Emalio[1,a], Hugues C. Nana-Djeunga[1,a], Cédric B. Chesnais[2], Sébastien D.S. Pion[2], Jules B. Tchatchueng-Mbougua[3], Michel Boussinesq[2], María-Gloria Basáñez[4,a,*], Joseph Kamgno[1,5,a,$]

[1]Centre for Research on Filariasis and other Tropical Diseases (CRFilMT), Yaoundé, Cameroon; [2]Institut de Recherche pour le Développement (IRD), UMI233/ INSERM U1175/ Université de Montpellier, 911 avenue Agropolis, BP 64501, 34394 Montpellier Cedex 5, France; [3]Service d'Epidémiologie, Centre Pasteur du Cameroun, Membre du Réseau International des Instituts Pasteur, Yaoundé, Cameroun; [4]MRC Centre for Global Infectious Disease Analysis and London Centre for Neglected Tropical Disease Research, Department of Infectious Disease Epidemiology, School of Public Health, Imperial College London, London, United Kingdom; [5]Faculty of Medicine and Biomedical Sciences, University of Yaoundé I, Cameroon

[a] Contributed equally to this manuscript

[*] **Corresponding Author:** Prof. M.G. Basáñez, MRC Centre for Global Infectious Disease Analysis and London Centre for Neglected Tropical Disease Research, Department of Infectious Disease Epidemiology, School of Public Health, Imperial College London, Norfolk Place, London W2 1PG, United Kingdom (m.basanez@imperial.ac.uk)

[$] **Alternative Corresponding Author:** Prof. J. Kamgno, Centre for Research on Filariasis and other Tropical Diseases (CRFilMT), P.O. Box 5797, Yaoundé, Cameroon (kamgno@crfilmt.org)

Supplementary Tables

Table S1. Characteristics of the 28 skin snip test (SST) false-positive individuals

No.	Health District	Community of residence	Sex	Age (yr)	*Loa loa* status	*Loa* MFD (mf/mL)	*Loa* MFD class (mf/mL)
1	Ndelele	Pana	F	7	Positive	11980	>4080
2	Mouloundou	Mikel	F	9	Negative	0	≤4080
3	Ndelele	Pana	F	9	Positive	5700	>4080
4	Mouloundou	Mikel	F	9	Negative	0	≤4080
5	Nguelemendouka	Azemkout	F	13	Positive	18700	>4080
6	Nguelemendouka	Azemkout	F	14	Negative	0	≤4080
7	Nguelemendouka	Azemkout	F	28	Positive	4840	>4080
8	Lomie	Adjela	F	38	Positive	54180	>4080
9	Kete	Timangolo	M	39	Positive	14000	>4080
10	Kete	Timangolo	F	40	Positive	600	≤4080
11	Ndelele	Pana	M	41	Positive	10720	>4080
12	Nguelemendouka	Azemkout	M	44	Positive	85200	>4080
13	Nguelemendouka	Azemkout	F	46	Positive	8320	>4080
14	Lomie	Adjela	F	50	Negative	0	≤4080
15	Ndelele	Pana	F	50	Positive	6180	>4080
16	Ndelele	Pana	M	51	Positive	12720	>4080
17	Kete	Timangolo	F	52	Positive	7440	>4080
18	Ndelele	Pana	M	54	Positive	13760	>4080
19	Kete	Timangolo	M	55	Positive	2600	≤4080
20	Mouloundou	Mikel	M	60	Positive	4240	>4080
21	Nguelemendouka	Azemkout	F	60	Positive	37620	>4080
22	Nguelemendouka	Azemkout	F	60	Positive	24140	>4080
23	Lomie	Azemkout	F	68	Negative	0	≤4080
24	Lomie	Adjela	F	68	Negative	0	≤4080
25	Nguelemendouka	Adjela	F	68	Positive	17200	>4080
26	Nguelemendouka	Azemkout	F	73	Positive	4180	>4080
27	Nguelemendouka	Azemkout	F	74	Positive	5480	>4080
28	Lomie	Adjela	F	83	Negative	0	≤4080

F: female; M: male; *Loa* MFD: *Loa loa* microfilarial density; mf: microfilariae; mL: milliliter.

Table S2. Multivariate association between skin snip test (SST) false positivity (for *Onchocerca volvulus*), sex and (fractional polynomial) of *Loa loa* microfilarial density (FP *Loa* MFD) among Ov16-negative individuals (n = 1,011)

Variables	Multivariate analysis			
	Coefficient	Standard error	z-value	*P*
Intercept	−2.202	0.249	−8.820	< .001
Sex				
Female				–
Male	−1.094	0.479	−2.280	.0226
FP (*Loa* MFD)	0.610	0.075	8.102	< .001

FP: fractional polynomial; *Loa* MFD: *Loa loa* microfilarial density; mf: microfilariae; mL: milliliter.

Table S3. Values of Akaike Information Criterion (AIC) and Deviance Information Criterion (DIC) for selection of best-fit logistic regression model

Model	AIC	DIC
Null	258.1	256.06
~ *Loa* MFD	230.53	226.53
~ Sex + *Loa* MFD	229.01	223.01
~ Sex + FP(*Loa* MFD)	183.4	177.40

Loa MFD: *Loa loa* microfilarial density (mf/mL); mf: microfilariae; mL: milliliter; FP: fractional polynomial; the FP order was equal to 0, and the fractional polynomial function was $\log\left(\frac{Loa\ \mathrm{MFD}+20}{1000}\right)$.

Table S4. Predicted probabilities (expressed as percent, %) of being skin snip test (SST) false positive (for *Onchocerca volvulus*) by sex for *Loa loa* microfilarial density (*Loa* MFD) values

	L. loa microfilarial density (*Loa* MFD) mf/mL									
	0	1000	2000	4080	10000	20000	30000	50000	70000	80000
Sex										
Female	1.0	10.1	14.5	20.7	31.1	40.8	46.9	54.6	59.7	61.6
Male	0.3	3.6	5.4	8.1	13.1	18.7	22.8	28.7	33.1	34.9
Ratio	3.3	2.8	2.7	2.6	2.4	2.2	2.1	1.9	1.8	1.8
Abs. diff.	0.7	6.5	9.1	12.6	18.0	22.1	24.1	25.9	26.6	26.7
Prop. diff.	2.3	1.8	1.7	1.6	1.4	1.2	1.1	0.9	0.8	0.8

Loa MFD: *Loa loa* microfilarial density (mf/mL); mf: microfilariae; mL: milliliter.

Ratio = probability of false SST positivity in females/probability of false SST positivity in males.

Abs. diff: Absolute difference = probability of false SST positivity in females – probability of false SST positivity in males.

Prop. diff: Proportional difference of females with respect to males = (probability of false SST positivity in females – probability of false SST positivity in males)/probability of false SST positivity in males.
